# Naturalist's Monthly Report

**Published:** 1812-01

**Authors:** 


### NATURALIST'S MONTHLY REPORT.

### NOVEMBER.

FREEZING MONTH.

The fields their verdure now resign,
The bleating flocks and lowing kine
Give o'er their former play;
The feather'd tribes forget the notes,
Which joyful strain'd their vocal throats,
To chaunt the matin lay.

THERE has hitherto been much less indication of the approach of winter than is usual at this late season of the year. The weather has been mild and open, except the frosty nights betwixt the 19th and 23d; but the quantity of rain that has fallen has been very great.

The prevailing winds have been those from south-west, west, north-west, and north; but chiefly from the two latter quarters. The only days during which I recollect the wind to have been easterly were the 8th, 22d, 23d, and 25th. We had fresh gales on the 1st, 4th, 11th, 12th, 13th, 14th, and 16th. Strong gales on the 15th, and violent storms, with squally weather, on the 2d and 5th,

With

With respect to rain; until the 19th of the month there was so incessant a succession of wet, that we had only two fine days during the whole time. But from the 19th to the 30th, (with the exception of the 25th,) we had fair weather. The four latter days were foggy.

In my last Report I stated that the hirundines had all taken their departure on the 17th, but in this I find myself to have been in error, as an immense flight of them were remarked by a friend of mine in this neighbourhood so late as the 23d; but after that day none were to be seen except a few stragglers.

November 3d. Two or three swallows were this morning remarked in flight, about the surface of the river. In my next month's Report it will be seen that several were observed so late as even the beginning of December.

November 4th. I observed a blind worm *(anguis fragilis)* lying dead by the side of the road. As a proof of the great mildness of the season, I was this day shewn an apple-blossom; and, a few days afterwards, a branch of lilac in bloom.

November 7th. In moist places the oaks continue still in verdure; but the foliage of almost all other trees is entirely gone. Many of the summer and autumnal plants are yet in flower. I remarked, amongst these, the red-flowered lychnis *(lychnis disica flore rubro)*, common tormentil *(tormentilla officinalis)*, soft-leaved crane's-bill *(geranium molle)*, ivy-leaved snap-dragon *(antirrhinum cymbalaria)*, and the common wall-flower.

November 11th. Woodcocks are scarce. Gulls frequent the rivers and fields in the inland parts of the country.

November 14th. The greater periwinkle *(vinca major)*, which usually flowers only during the months of April and May, is now in great beauty in sheltered gardens in this neighbourhood. Sweet-scented violets are likewise in flower.

November 20th. The mulberry-trees continued in verdure till last night; but this morning, in consequence of the intensity of the frost, the leaves are all fallen.

November 22d. Large flocks of fieldfares begin to arrive.

Moles continue to throw up the earth, the frost not having yet penetrated the surface of the ground to a depth sufficient to prevent them from continuing their usual operations. I have not seen any species of bats flitting about for several evenings past.

The flowers of the strawberry-tree *(arbutus unedo)* fall.

November 27th and 28th. A considerable quantity of silver whiting *(gadus merlangus)*, of a size much greater than I have usually seen them, have been caught off this coast, with lines, some of them weighed upwards of two pounds and a half each.

November 30th. This evening a few mackrel were brought to shore in the herring nets. The herring fishers have been extremely successful. In no season, for many years back, has such a quantity of these fish been caught as this year; and the abundant supply has been peculiarly acceptable to the poor, many of whom salt and hang them up in their cottages for future subsistence.

*Hampshire.*

